# Supplementary material for: Physiological, Agronomical, and Proteomic Studies Reveal Crucial Players in Rice Nitrogen Use Efficiency under Low Nitrogen Supply
Source: Int J Mol Sci. 2022 Jun 8;23(12):6410. doi: 10.3390/ijms23126410 (PMC9224494; doi:10.3390/ijms23126410)
Supplement: Supplementary file 1 [file ijms-23-06410-s001.zip › Supplementary figures.pdf]

Supplementary Figures

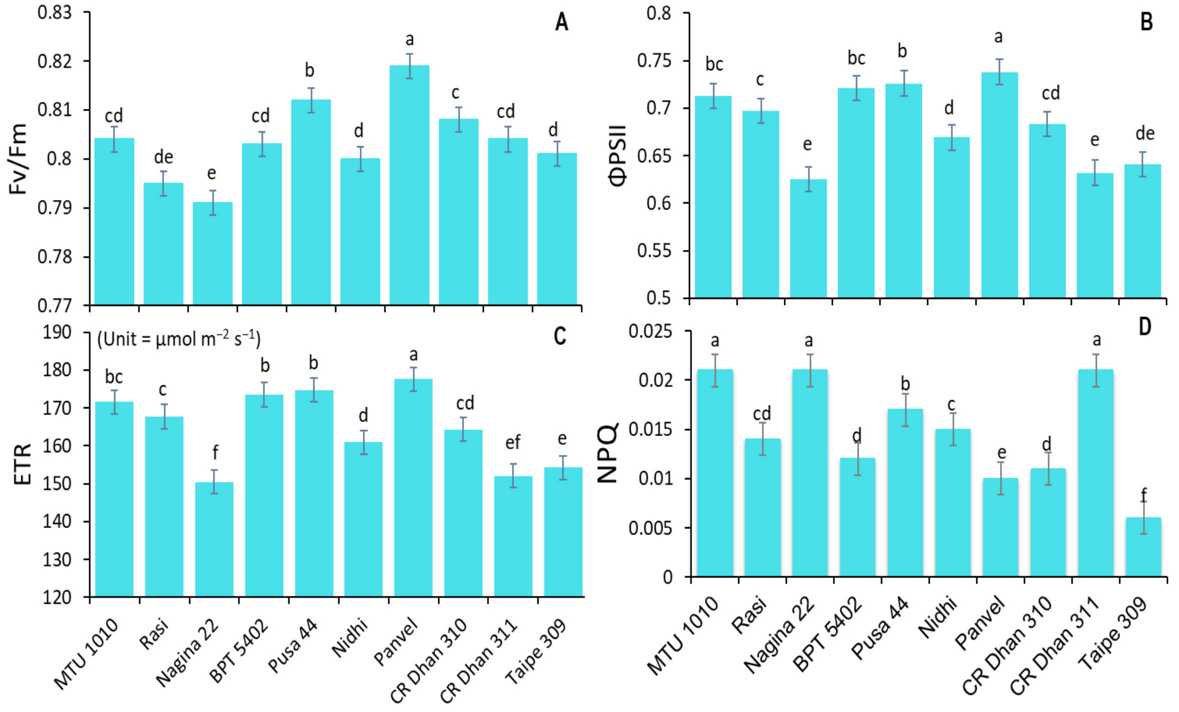

Supplementary Figure S1. Measurement of chlorophyll fluorescence of photosystem II of ten rice cultivars at low-NPS supply. (A) Total photochemical quantum efficiency, (B) effective photochemical quantum efficiency, (C) electron transport rate, and (D) non-photochemical quenching. Graph bars are representing mean ± SE with different letters denoting significant difference at $P < 0.05$.

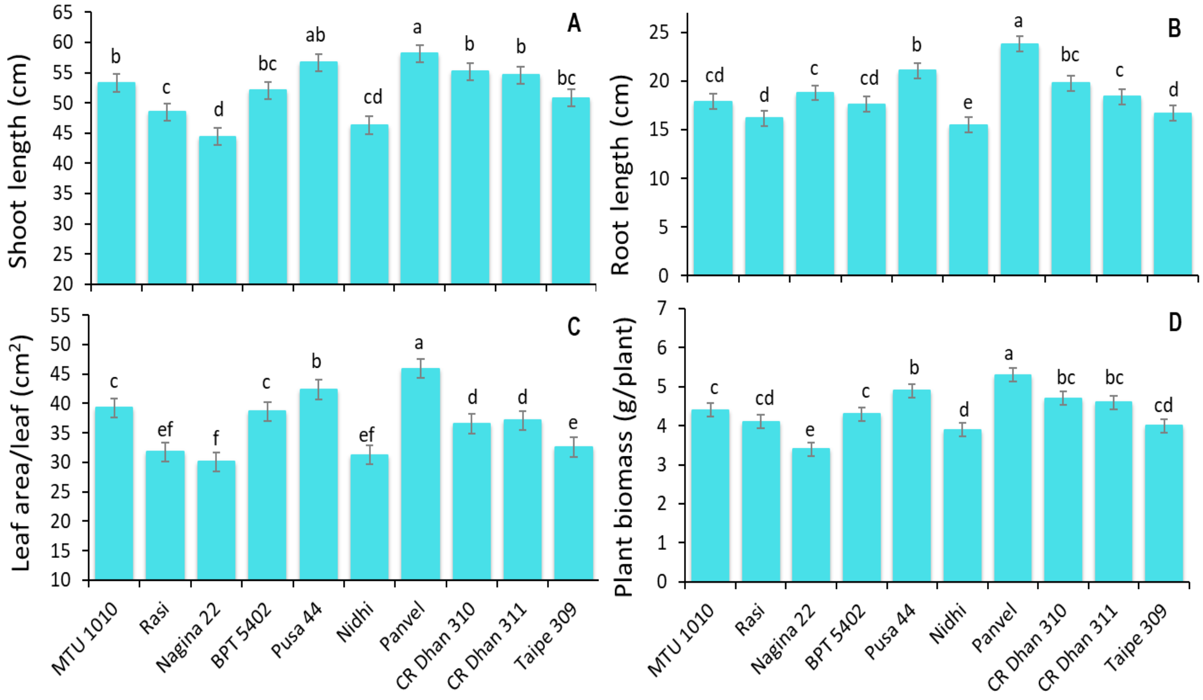

Supplementary Figure S2. Measurement of plant growth traits of ten rice cultivars at low-NPS supply during screening. (A) Shoot and (B) root lengths were measured in centimeter by measuring-scale, (C) leaf area of 5th leaf was calculated in centimeter square and, (d) plant biomass was measured in gram per plant. Graph bars are representing mean ± SE with different letters denoting significant difference at < 0.05.

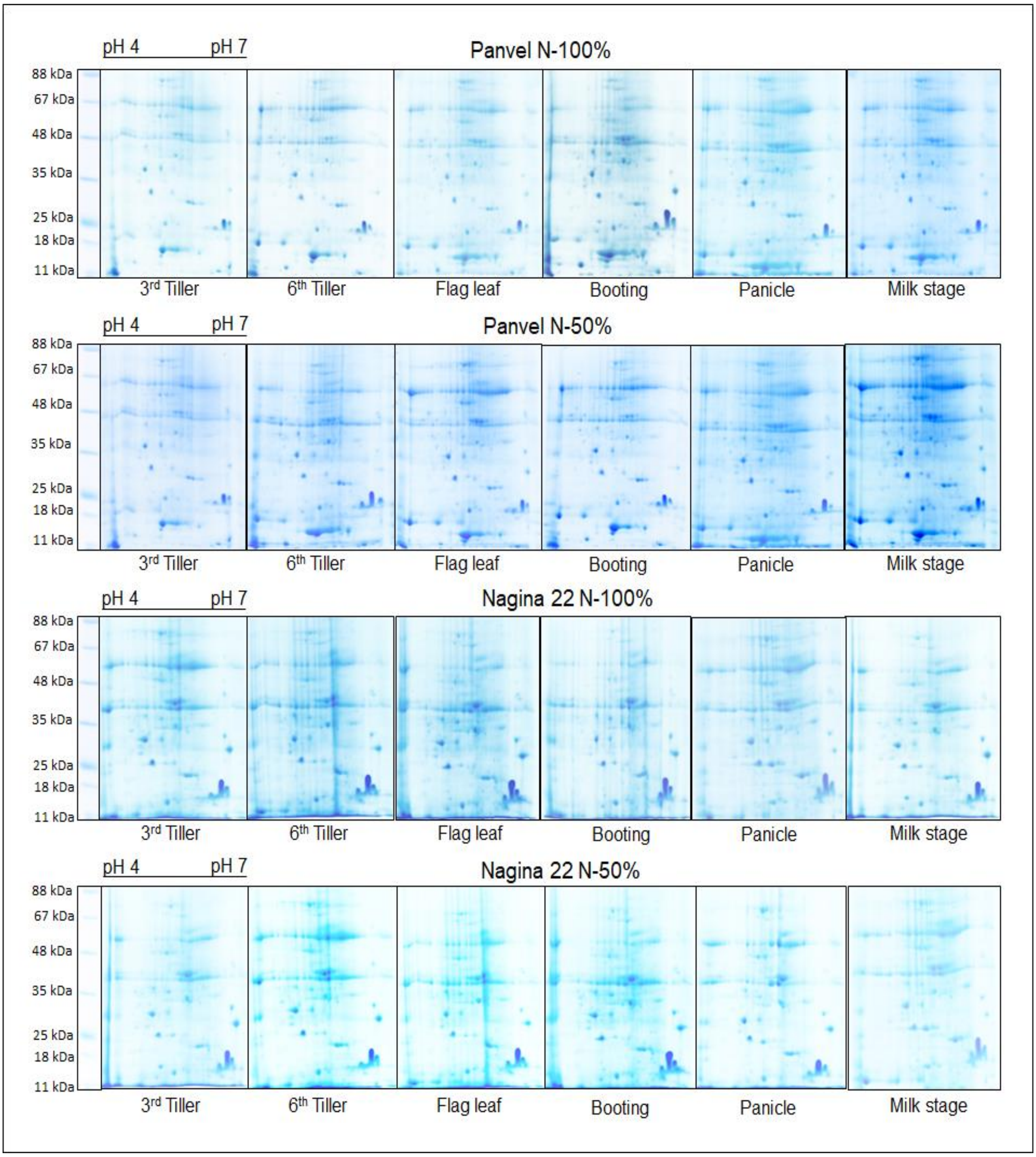

Supplementary Figure S3. Representation of 2-DE maps of leaf proteome of six specific growth stages of rice cultivar Panvel and Nagina 22 at optimum (100%) and low (50%) nitrogen supply.
