# Supplementary material for: Physiological, Agronomical, and Proteomic Studies Reveal Crucial Players in Rice Nitrogen Use Efficiency under Low Nitrogen Supply
Source: Int J Mol Sci. 2022 Jun 8;23(12):6410. doi: 10.3390/ijms23126410 (PMC9224494; doi:10.3390/ijms23126410)
Supplement: Supplementary file 1 [file ijms-23-06410-s001.zip › Supplementary tables S1-S7, S9.pdf]

Supplementary Tables

Supplementary Table S1. One-way ANOVA analysis of gas-exchange, SPAD, chlorophyll fluorescence, plant growth, Rubisco activity, and leaf mineral nutrient content traits of rice cultivars

Parameter	***F*-Value**	***P*-Value**	R-sq
P_n	4.57	0.002	67.27%
g_s	3.90	0.005	63.73%
C_i	3.57	0.007	59.21%
T_r	1.63	0.174	42.30%
SPAD	4.37	0.003	66.31%
F	1.77	0.197	39.48%
F_o’	2.85	0.043	51.22%
F_m’	2.91	0.025	56.03%
F_v/F_m	11.21	0.000	48.37%
Φ_{PSII}	7.53	0.000	39.88%
ETR	4.48	0.001	40.12%
qN	5.24	0.008	61.89%
qP	8.62	0.000	55.26%
qL	3.63	0.003	47.66%
NPQ	9.31	0.001	61.49%
Y(NO)	4.47	0.002	57.71%
Y(NPQ)	3.88	0.005	49.92%
Shoot length	4.36	0.000	37.54%
Root length	5.44	0.003	44.67%
Leaf area	7.86	0.000	52.38%
Plant biomass	3.77	0.001	62.55%
Rubisco enzyme activity	4.45	0.000	60.93%
Rubisco protein content	3.89	0.003	49.88%
Soluble protein content	7.65	0.000	32.58%
Leaf N content	10.13	0.000	55.61%
Leaf P content	4.12	0.021	61.78%
Leaf S content	3.72	0.033	59.25%

Supplementary Table S2. Gas-exchange and SPAD measurements of ten rice cultivars under low NPS in the screening experiment

Cultivars	P_n (μ mol m^{-2} s^{-1})	g_s (mol m^{-2} s^{-1})	C_i (μ mol CO_2 mol^{-1})	T_r (mmol m^{-2} s^{-1})	SPAD (nmol chl. cm^{-2})
MTU 1010	22.24 ± 1.76 ^{c}	0.331 ± 0.023 ^{d}	238 ± 14.52 ^{bc}	13.6 ± 1.02 ^{c}	47.01 ± 3.41 ^{b}
Rasi	21.22 ± 1.44 ^{de}	0.323 ± 0.021 ^{e}	231 ± 12.34 ^{cd}	13.1 ± 1.07 ^{cd}	45.42 ± 4.22 ^{c}
Nagina 22	19.68 ± 1.51 ^{e}	0.317 ± 0.022 ^{f}	227 ± 14.28 ^{d}	12.7 ± 1.09 ^{d}	43.81 ± 3.82 ^{d}
BPT 5402	22.09 ± 1.13 ^{cd}	0.328 ± 0.024 ^{de}	235 ± 11.93 ^{c}	13.5 ± 1.01 ^{c}	46.62 ± 3.39 ^{bc}
Pusa 44	23.11 ± 1.56 ^{b}	0.344 ± 0.026 ^{b}	245 ± 13.32 ^{b}	14.2 ± 1.05 ^{b}	48.11 ± 4.21 ^{ab}
Nidhi	21.57 ± 2.23 ^{d}	0.324 ± 0.021 ^{e}	230 ± 12.37 ^{cd}	13.2 ± 1.02 ^{cd}	45.74 ± 2.97 ^{c}
Panvel	24.21 ± 2.12 ^{a}	0.352 ± 0.031 ^{a}	252 ± 15.78 ^{a}	15.6 ± 1.12 ^{a}	49.54 ± 3.65 ^{a}
CR Dhan 310	22.94 ± 2.45 ^{bc}	0.339 ± 0.025 ^{c}	243 ± 16.22 ^{bc}	14.3 ± 1.11 ^{b}	47.47 ± 4.11 ^{b}
CR Dhan 311	22.92 ± 1.53 ^{bc}	0.337 ± 0.028 ^{cd}	241 ± 12.71 ^{bc}	13.8 ± 1.10 ^{bc}	47.29 ± 4.23 ^{b}
Taipe 309	21.98 ± 2.03 ^{cd}	0.326 ± 0.024 ^{de}	232 ± 13.15 ^{c}	13.3 ± 1.06 ^{cd}	46.36 ± 3.74 ^{bc}

Note: Result data are the mean ± SE and the different letters indicate statistically significant differences among cultivars in Tukey comparisons ($P < 0.05$). P_n = net photosynthetic rate; g_s = stomatal conductance; C_i = intercellular CO2 rate; T_r = transpiration rate; and SPAD = soil and plant analyzer development.

Supplementary Table S3. Coefficient and yield of photochemical and non-photochemical quenching of photosystem II of the ten rice cultivars in the screening experiment

Cultivars	qN	qP	qL	Y(NO)	Y(NPQ)
MTU 1010	0.024 ± 0.006 ^{ab}	0.781 ± 0.045 ^{e}	0.415 ±0.039 ^{e}	0.367 ± 0.021 ^{a}	0.008 ± 0.002 ^{a}
Rasi	0.016 ± 0.003 ^{bc}	0.931 ± 0.076 ^{a}	0.737 ± 0.048 ^{a}	0.259 ± 0.019 ^{e}	0.003 ± 0.001 ^{c}
Nagina 22	0.025 ± 0.007 ^{a}	0.813 ± 0.037 ^{d}	0.479 ± 0.021 ^{d}	0.352 ± 0.034 ^{b}	0.007 ± 0.003 ^{ab}
BPT 5402	0.014 ± 0.003 ^{c}	0.900 ± 0.068 ^{b}	0.643 ± 0.031 ^{b}	0.276 ± 0.021 ^{de}	0.003 ± 0.001 ^{c}
Pusa 44	0.019 ± 0.005 ^{b}	0.896 ± 0.043 ^{bc}	0.621 ± 0.033 ^{bc}	0.270 ± 0.018 ^{de}	0.004 ± 0.001 ^{bc}
Nidhi	0.018 ± 0.004 ^{b}	0.839 ± 0.039 ^{cd}	0.512 ± 0.027 ^{c}	0.326 ± 0.032 ^{bc}	0.005 ± 0.002 ^{b}
Panvel	0.012 ± 0.002 ^{cd}	0.853 ± 0.044 ^{c}	0.515 ± 0.026 ^{c}	0.300 ± 0.027 ^{cd}	0.003 ± 0.001 ^{c}
CR Dhan 310	0.013 ± 0.003 ^{cd}	0.847 ± 0.048 ^{c}	0.517 ± 0.036 ^{c}	0.314 ± 0.024 ^{c}	0.003 ± 0.001 ^{c}
CR Dhan 311	0.024 ± 0.007 ^{ab}	0.781 ± 0.033 ^{e}	0.415 ± 0.026 ^{e}	0.367 ± 0.031 ^{a}	0.008 ± 0.003 ^{a}
Taipe 309	0.007 ± 0.002 ^{e}	0.891 ± 0.041 ^{bc}	0.620 ± 0.038 ^{bc}	0.285 ± 0.022 ^{d}	0.002 ± 0.001 ^{cd}

Note: Result data are the mean ± SE and the different letters indicate statistically significant differences among cultivars in Tukey comparisons (P < 0.05). qN = Coefficient of non-photochemical fluorescence quenching; qP and qL = Coefficient of photochemical fluorescence quenching; Y(NO) = Quantum yield of non-regulated fluorescence emission; and Y(NPQ) = Quantum yield non-photochemical fluorescence quenching.

Supplementary Table S4. Rubisco enzyme activity, Rubisco protein content and, the soluble protein content of leaves of ten rice cultivars in the screening experiment

Cultivars	**Rubisco enzyme activity** (nmol^{-1} g^{-1} FW)	**Rubisco protein content** (mg cm^{-1} FW)	**Soluble protein content** (mg g^{-1} FW)
MTU 1010	209.13 ± 9.22 ^{bc}	0.138 ± 0.021 ^{cd}	29.22 ± 3.42 ^{c}
Rasi	189.71 ± 7.54 ^{e}	0.132 ± 0.023 ^{de}	23.56 ± 2.99 ^{de}
Nagina 22	187.65 ± 7.11 ^{e}	0.129 ± 0.016 ^{e}	21.79 ± 2.89 ^{e}
BPT 5402	197.55 ± 5.93 ^{d}	0.136 ± 0.023 ^{d}	27.55 ± 3.67 ^{cd}
Pusa 44	211.36 ± 6.27 ^{b}	0.145 ± 0.022 ^{b}	30.12 ± 4.57 ^{b}
Nidhi	193.81 ± 5.48 ^{de}	0.131 ± 0.019 ^{e}	24.68 ± 3.22 ^{d}
Panvel	224.62 ± 7.89 ^{a}	0.148 ± 0.026 ^{a}	33.57 ± 4.12 ^{a}
CR Dhan 310	203.72 ± 6.93 ^{c}	0.142 ± 0.021 ^{c}	28.44 ± 3.25 ^{cd}
CR Dhan 311	205.28 ± 6.22 ^{c}	0.141 ± 0.024 ^{c}	29.12 ± 3.71 ^{c}
Taipe 309	195.47 ± 5.14 ^{de}	0.134 ± 0.022 ^{de}	24.11 ± 3.00 ^{d}

Note: Result data are the mean ± SE (FW = fresh weight) and the different letters indicate statistically significant differences among cultivars in Tukey comparisons (P < 0.05).

Supplementary Table S5. Leaf nitrogen, phosphorus, and sulphur contents of the ten rice cultivars grown at the low-NPS supply

**Cultivars**	**Leaf N content** (mg g^{-1} DW)	**Leaf P content** (mg g^{-1} DW)	**Leaf S content** (mg g^{-1} DW)
MTU 1010	4.20 ± 0.13 ^{c}	2.05 ± 0.11 ^{bc}	2.91 ± 0.22 ^{ab}
Rasi	3.92 ± 0.14 ^{e}	1.72 ± 0.09 ^{de}	2.65 ± 0.18 ^{c}
Nagina 22	3.12 ± 0.09 ^{f}	1.61 ± 0.07 ^{e}	2.24 ± 0.15 ^{d}
BPT 5402	4.16 ± 0.12 ^{cd}	1.84 ± 0.10 ^{d}	2.72 ± 0.16 ^{bc}
Pusa 44	4.44 ± 0.11 ^{b}	2.12 ± 0.12 ^{b}	2.83 ± 0.19 ^{b}
Nidhi	4.10 ± 0.13 ^{d}	1.81 ± 0.11 ^{d}	2.66 ± 0.14 ^{c}
Panvel	4.60 ± 0.16 ^{a}	2.33 ± 0.14 ^{a}	3.04 ± 0.21 ^{a}
CR Dhan 310	4.32 ± 0.11 ^{bc}	1.95 ± 0.10 ^{c}	2.80 ± 0.20 ^{b}
CR Dhan 311	4.28 ± 0.13 ^{bc}	1.92 ± 0.11 ^{c}	2.81 ± 0.17 ^{b}
Taipe 309	4.11 ± 0.10 ^{d}	1.80 ± 0.12 ^{d}	2.61 ± 0.11 ^{c}

Note: Result data are the mean ± SE (DW = dry weight) and the different letters indicate statistically significant differences among cultivars in Tukey comparisons ($P < 0.05$). N = nitrogen; P = phosphorus; and S = sulphur.

Supplementary Table S6. Analysis of variance (3-way ANOVA) of gas-exchange, chlorophyll fluorescence, biochemical, and plant growth traits of the rice cultivars grown under two N levels

**Traits**	**C**	**T**	**GS**	**C × T**	**C × GS**	**T × GS**	**C × T × GS**	R^2
P_n	9.56*	35.66***	28.92**	29.34**	24.75*	52.61**	1.63*	69.74%
g_s	6.89*	47.84**	31.22*	31.87*	27.62*	42.57*	0.78^{ns}	58.47%
C_i	10.22**	43.53**	35.21*	33.26*	29.44^{ns}	39.56*	0.81^{ns}	48.34%
SPAD	8.12*	35.69*	51.22**	27.54*	42.33*	39.46*	1.52^{ns}	55.49%
F_v/F_m	3.16*	14.86***	23.41**	11.45**	12.32*	17.13*	1.77*	49.83%
Φ_{PSII}	2.67**	16.47**	10.56*	9.86*	7.94*	13.52*	1.66*	55.38%
ETR	21.24**	32.45**	18.74*	24.33*	19.52*	14.22*	1.92*	63.77%
qP	4.55*	28.94**	21.44*	14.57*	7.66^{ns}	11.87*	0.89^{ns}	52.62%
NPQ	7.23*	31.66***	22.91**	21.37**	14.11*	25.47**	1.97*	58.43%
Shoot length	15.78**	42.57***	37.58**	25.48**	21.18*	28.35**	2.33*	65.21%
Leaf area	8.67*	41.32**	29.69*	26.47*	17.88*	29.45*	0.85^{ns}	51.36%
Plant biomass	7.84*	22.43**	31.65**	17.86*	19.72*	23.19*	1.93*	49.71%
Rubisco enzyme activity	12.28*	37.64***	35.48**	21.17*	20.83*	25.66*	2.76*	62.57%
Rubisco protein content	5.42*	25.49**	19.63*	18.38*	12.55^{ns}	18.82*	0.71^{ns}	38.95%
Soluble protein content	10.33*	31.53**	22.47*	19.77*	15.92*	23.41*	0.79^{ns}	44.95%
Leaf N content	6.43*	42.58***	19.62**	24.81**	12.48*	17.99*	1.83*	47.82%

Note: The values in each column represents *F*-value and asterisks denotes the significant differences at $*P < 0.05$, $**P < 0.01$ and $***P < 0.001$. C = cultivars; T = treatment; and ns = not significant.

Supplementary Table S7. Analysis of variance (2-way ANOVA) of total tillers per hill, agronomic traits, N uptake efficiency, N use efficiency

Traits	C	T	C × T	R^2
Total tillers per hill	17.48***	35.73***	2.14*	64.82%
Panicles per hill	9.39**	43.82***	2.73*	71.36%
Panicle length	12.23**	52.14**	1.92*	56.38%
Grains per panicle	23.45**	46.71***	2.67*	49.63%
1000gains weight	6.26*	50.42**	1.18^{ns}	43.85%
Panicle per meter square	12.66**	34.85***	2.88*	51.28%
Grain yield	22.59***	44.84***	1.85*	48.52%
N uptake efficiency	9.78**	47.16***	1.86*	53.47%
N utilization efficiency	15.87**	45.93***	2.21*	67.19%
N use efficiency	13.54**	42.19***	2.01*	62.33%

Note: The values in each column represents *F*-value and asterisks denotes the significant differences at *$P < 0.05$, **$P < 0.01$ and ***$P < 0.001$. C = cultivars; T = treatment; and ns = not significant.

Supplementary Table S9. List of genes for validation with their forward and reverse primers

Accession number	Protein description	Gene name	Primers [F = Forward; R = Reverse]
Q7F2G3	Carbonic anhydrase	Os01g0639900	F 5´-TGGAGAACCTCAAGACCTAC-3´<br>R 5´-ACTGACGAACTGAACGGAC-3´
Q0DG05	Photosystem I reaction center subunit VI	PSAH	F 5´-AGGATGTTATGCTCTGCCTG-3´<br>R 5´-GCTGCTACTGCTGTTTTCTTG-3´
Q10HD0	Chlorophyll a-b binding protein, chloroplastic	RCABP89	F 5´-CACCACACACCACAAAAAAC-3´<br>R 5´-AGGTCCATACCAGATGCTC-3´
Q7XCK0	Glutathione S-transferase	GST	F 5´-CCTGTCTGTCAAACTGCTTTC-3´<br>R 5´-ACCTTCCTATTCATCTTCCCC-3´
P16081	Nitrate reductase [NADH] 1	NIA1	F 5´-CGAGTCCGACAACTACTACC-3´<br>R 5´-TCACCGAGTTCACGTTCAG-3´
Q5W6H5	Chlorophyll synthase, chloroplastic	CHLG	F 5´-ATGAAAGCAGCAATCGCC-3´<br>R 5´-GCAAAGTAGCAAAACGACCC-3´
Q0IU70	Nitrogen fixation protein, putative	Os11g07916	F 5´-ATGAAAGCAGCAATCGCC-3´<br>R 5´-GCAAAGTAGCAAAACGACCC-3´
Q6AUR2	Nitrogen regulatory protein P-II homolog	GLB	F 5´-TGAAAAGGCAAGAACAGGAG-3´<br>R 5´-CATCATCACTAATCAACAGGGG-3´
Q0JG75	Photosystem II reaction center PSB28 protein	PSB28	F 5´-TTTTTCTCCTTTTTTCCCCCTC-3´<br>R 5´-GCACACACCAAACAACTCATC-3´
P93431	RuBisCO activase	RCA	F 5´-GATGTAACACACAGAGTGCC-3´<br>R 5´-ACCACCAATCTCTTCTACCC-3´
Q10DV7	Actin-1 (as reference gene)	ACT1	F 5´-TGACAGGATGAGCAAGGAG-3´<br>R 5´-CAAAAAGAGAGAAACAAGCAGG-3´
